# Advances of Glycometabolism Engineering in Chinese Hamster Ovary Cells

**DOI:** 10.3389/fbioe.2021.774175

**Published:** 2021-12-02

**Authors:** Huan-Yu Zhang, Zhen-Lin Fan, Tian-Yun Wang

**Affiliations:** ^1^ Department of Biochemistry and Molecular Biology, Xinxiang Medical University, Xinxiang, China; ^2^ International Joint Research Laboratory for Recombinant Pharmaceutical Protein Expression System of Henan, Xinxiang, China; ^3^ Institutes of Health Central Plain, Xinxiang Medical University, Xinxiang, China

**Keywords:** CHO cells, glycometabolism engineering, pyruvate metabolism, aerobic oxidation of glucose, metabolic models

## Abstract

As the most widely used mammalian cell line, Chinese hamster ovary (CHO) cells can express various recombinant proteins with a post translational modification pattern similar to that of the proteins from human cells. During industrial production, cells need large amounts of ATP to support growth and protein expression, and since glycometabolism is the main source of ATP for cells, protein production partly depends on the efficiency of glycometabolism. And efficient glycometabolism allows less glucose uptake by cells, reducing production costs, and providing a better mammalian production platform for recombinant protein expression. In the present study, a series of progresses on the comprehensive optimization in CHO cells by glycometabolism strategy were reviewed, including carbohydrate intake, pyruvate metabolism and mitochondrial metabolism. We analyzed the effects of gene regulation in the upstream and downstream of the glucose metabolism pathway on cell’s growth and protein expression. And we also pointed out the latest metabolic studies that are potentially applicable on CHO cells. In the end, we elaborated the application of metabolic models in the study of CHO cell metabolism.

## Introduction

In recent years, the proportion of biological drugs in the global pharmaceutical market has been expanding. Since 2002, more than 300 biological drugs have been approved by the FDA (Food and Drug Administration, 2004; Tihanyi and Nyitray, 2021), and the number continues to grow.

For macromolecular complex recombinant therapeutic proteins (RTPs), proper folding and post-translational modifications of proteins are required to meet their biological activity; therefore, mammalian cells are often used to produce RTPs. Among the mammalian cells, such as mouse myeloma cells, mouse fibroblasts, human embryonic kidney 293 cells, small hamster kidney cells, and human retina-derived PerC6 cells (Bebbington et al., 1992; Barnes et al., 2001; Jones et al., 2003; Griffin et al., 2007; Baldi et al., 2010), Chinese hamster ovary (CHO) cells are the most widely used mammalian cell line, and nearly 70% of RTPs are produced using this system (Cheung et al., 2016). The CHO expression system has several advantages over other expression systems: 1) It is capable of both appressed growth and high cell density suspension culture in special media, which facilitates large-scale industrial production (Lai et al., 2013). 2) Almost no human virus can multiply in CHO cells, suggesting that it is potentially less dangerous (Boeger et al., 2005). 3) The expressed proteins are closest to natural proteins in terms of molecular structure, physicochemical properties, and biological functions, and the glycosylation of CHO cell-expressed proteins is closer to that of human-derived cells due to the lack of immunogenic α-galactose epitopes (Ghaderi et al., 2012). 4) CHO cells are fibroblasts with low endogenous protein secretion, which facilitates the isolation and purification of recombinant proteins (Mohan et al., 2008), 5) and can efficiently amplify and express exogenous genes (Li et al., 2018).

Since the first RTP, tissue plasminogen activator (tPA), was approved for marketing in 1986 (Kaufman et al., 1985), CHO cells have been the cell line of choice for expressing RTPs as their expression system. Protein production has exceeded 10 g/L due to the optimization of culture medium and the development of production culture processes (Kim et al., 2012). But the application of cell engineering technology is promising to achieve a breakthrough in yield.

To meet the growing market demand for biopharmaceuticals, how to continuously innovate manufacturing processes to achieve higher volume productivity in shorter time, as well as stable product quality and lower production cost is a hot research topic in biopharmaceutical field nowadays. To achieve this, researchers overexpressed beneficial genes or repressed disadvantageous genes by genomic knock-out or siRNA-mediated knock-down to improve performance of CHO manufacturing cell lines. These cell engineering approaches classically focused on the cellular growth, metabolism, apoptosis, the protein glycosylation, secretion, and production (Le Fourn et al., 2014; Yang et al., 2015). We also constructed DNA methyltransferase-deficient (Dnmt3a-deficient) CHO cells to reduce DNA methylation (Jia et al., 2018; Wang et al., 2019). Then we realized that metabolic engineering strategies targeting key enzymes in the glucose metabolism of CHO cells and the enzymes associated with key enzyme activities can further optimize the cell lines to facilitate industrial production.

This paper reviews the progress of research on optimizing CHO cellular glucose metabolism in three aspects: alternative carbon sources, pyruvate metabolism, and mitochondrial metabolism using gene editing techniques.

## CHO Cell Glucose Metabolism Pathway

Glucose is the main component of mammalian cell culture medium and the main carbon source material that provides energy for cell growth. There are four main metabolic pathways of glucose in CHO cells: the conversion to lactic acid through the glycolytic pathway, which provides energy for cell growth; the complete oxidation to CO_2_ by entering the tricarboxylic acid cycle (TCA cycle) continuously; the conversion to ribose phosphate through the pentose phosphate pathway, which is used for the generation of nucleic acid; and the synthesis to other substances such as amino acids and fatty acids.

**FIGURE 1:**
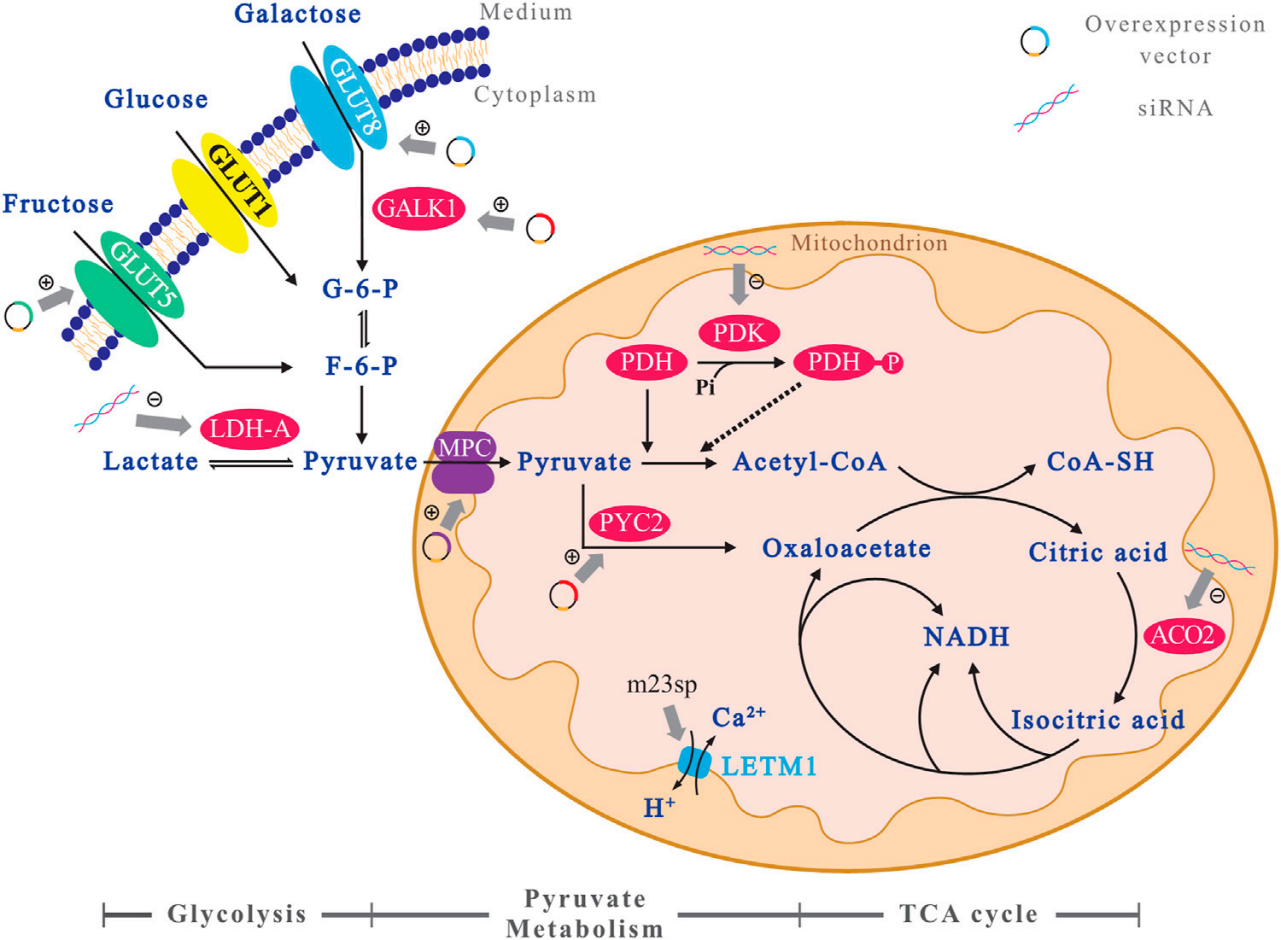
A profile of the GOIs’ sites in the glucose metabolism pathway of CHO cells. To better demonstrate the relationship between the target genes of metabolic engineering and the glucose metabolism pathway in CHO cells, an illustration is presented. The GOIs which are overexpressed have been marked with “+,” and the others which are down-expressed *via* siRNA have been marked with “−.” All the GOIs are indicated using background of colorful circles, and the metabolic pathways are marked as an axis below the figure.

It has been shown that glucose in cultured mammalian cells produces ATP mainly through the glycolytic pathway, with 2 mol of ATP per mole of glucose, but 36 mol of ATP per mole of glucose if it can be fully oxidized to CO_2_ in the TCA cycle (Berg et al., 2007). It can be seen that the energy generation efficiency of the TCA cycle is much higher than that of the glycolysis. However, the isotopic tracer method analysis revealed that more than 95% of glucose is converted to lactate through the glycolysis pathway, 3.6% of glucose enters the pentose phosphate pathway, and only 0.6% are carried into the TCA cycle (Petch and Butler, 1994). This shows that, despite the enormous potential of established cell lines, CHO cells have inherent metabolic limitations: High glycolytic rate even in the presence of oxygen. This is common to the Warburg effect found in tumor cells (Bulte et al., 2020). To improve the metabolic efficiency of CHO cells, researchers have regulated the expression of several genes of interest (GOIs) in the glucose metabolism pathway (Figure 1). And the effects of these glycometabolism engineering methods on cell culture and protein production of CHO cells are listed in Table 1.

**TABLE 1:** The effects of glycometabolism engineering methods on CHO cells.

Metabolic pathway	Target gene	Gene function	Gene regulation strategy	Cell culture	Protein production	References
Carbohydrate intake	GALK1	Galactose → G-6-P	Overexpression	39% increase in specific growth rate; 54% increase in ΔL/ΔGal	—	Jimenez et al. (2019)
	GLUT8	Transport galactose	Overexpression	Increase growth rate	—	Jiménez et al. (2011)
	GLUT5	Transport fructose	Overexpression	Enable cells to metabolize fructose in late growth	—	Le et al. (2013)
Pyruvate metabolism	MPC	Transport pyruvate	Overexpression	Increase live cell density by up to approximately 1.9 times; reduce lactate production by 50%	Increase alkaline phosphatase and monoclonal antibody production by 40%	Bulte et al. (2020)
	PDK	Phosphorylates PDH	Down-expression *via* siRNA	Increase PDH activity; facilitate pyruvate entry into the TCA cycle	—	Zhou et al. (2011)
	LDH-A	Pyruvate ↔ Lactate	Down-expression *via* siRNA	Reduce lactate production by 45–79%	No increase in specific productivity and protein production	Kim and Lee (2007)
	PYC2	Pyruvate → Oxaloacetate	Overexpression	Promote lactic acid consumption and reduce lactic acid accumulation by about four times	Increase monoclonal antibody production by 70%	Gupta et al. (2017)
TCA cycle	ACO2	Citric acid → Isocitric acid	Down-expression *via* siRNA	Significantly inhibit cell growth	—	Dhami et al. (2018)
	LETM1	Transport Ca^2+^	miR-23 sponge deplete miR-23	No increase in cell growth	Increase specific productivity and SEAP volume productivity for three times	Kelly et al. (2015)
	NAMPT	NAM → NMN	Potential application in CHO cells			Mori et al. (2014); Opitz and Heiland (2015)
	SLC25A51	Transport NAD^+^	Potential application in CHO cells			Girardi et al. (2020); Kory et al. (2020); Luongo et al. (2020)
	NMNAT	NMN → NAD^+^	Potential application in CHO cells			Croft et al. (2020)

## Alternative Carbon Sources

Various intrinsic (genetic) and external (environmental) factors act together during cell lineage development, among which environmental factors include the provision of key components, such as amino acids, carbohydrates, and metabolites, which promote cell growth, prolong culture viability, and increase productivity by supporting efficient cell metabolism. However, high provision of nutrients can increase the production and accumulation of metabolic wastes, such as lactate and ammonia, inhibiting normal cellular metabolism. Moreover, very rapid glycolysis can lead to the accumulation of pyruvate, which in turn generates lactate. In addition, the production of lactate and ammonia can further lead to inefficient metabolism, resulting in reduced cell growth and protein synthesis (Glacken et al., 1986; Kurano et al., 1990). Therefore, reducing the synthesis of toxic products such as ammonia and lactate is one of the main approaches to improve CHO culture. Considering the relationship between metabolism, cell growth, and recombinant protein production, metabolism-related genes are common GOIs for genetic engineering (Templeton et al., 2013). Altering the expression of key genes in the central carbon metabolism and the use of alternative carbon sources are beneficial to reduce lactate production to optimize the cell culture (Altamirano et al., 2013; Richelle and Lewis, 2017). Therefore, the strategy of using key genes of glucose metabolism as GOIs for metabolic engineering of CHO cells and simultaneously pairing them with the use of alternative carbon sources to culture CHO cells have been heavily studied and applied.

### Galactose

CHO cells cultured with galactose, instead of glucose as the sole carbon source, showed a low survival rate (Neermann and Wagner, 1996). In contrast, when cultured in a medium containing both glucose and galactose, the cells could utilize lactate and reduce lactate generation, probably because CHO cells metabolize galactose slower than glucose, reducing the glycolytic rate and avoiding a large accumulation of pyruvate. However, in this case, the galactose-related metabolism, which is much slower than that of glucose, also causes a decrease in the specific cell growth rate (Kelly et al., 2018). To improve galactose metabolism, CHO cells overexpressing galactose kinase (GALK1) were cultured in a medium containing galactose, which increased their specific growth rate by 39% and maintained their growth with galactose as the main carbon source. The slightly lower density of cells overexpressing GALK1 compared to controls may be related to metabolic stress due to GALK1 overexpression (Gu et al., 1995); however, lactate accumulation was less and the parameter ΔL/ΔGal increased by 54% indicating improved cellular galactose metabolism (Jimenez et al., 2019). Metabolic flux analysis during the glucose depletion phase versus the galactose depletion phase in CHO cells overexpressing GALK1 and control CHO cells showed that inefficient glucose metabolism led to pyruvate accumulation during the glucose depletion phase. This excess pyruvate is directed to the synthesis of alanine and lactate, which is characteristic of cells cultured with glucose as a carbon source (Altamirano et al., 2006; Wilkens et al., 2011). Furthermore, cells overexpressing GALK1 exhibited a lower glycolytic flux during the glucose depletion phase and a higher TCA cycle flux during the galactose depletion phase than the controls (Jimenez et al., 2019). Moreover, culturing CHO cells using galactose increases the sialic acid glycosylation modification of recombinant proteins (Liu et al., 2015). However, when CHO cells were cultured in a galactose medium, some amino acids, such as histidine and glutamine, were depleted in the early stages of cell growth and became growth limiting factors. Therefore, this method of culturing CHO cells with galactose as a carbon source can still be improved. In addition, overexpression of galactose transporter protein (GLUT8) can also increase the uptake and metabolism of galactose by cells (Jiménez et al., 2011).

### Fructose

The affinity of fructose transporter protein (GLUT5) for fructose is lower than that of glucose transporter protein (GLUT1) for glucose. Thus, replacing glucose in the medium with fructose while CHO cells express GLUT5 can also reduce lactate accumulation by reducing the glycolytic flux (Wlaschin and Hu, 2007). CHO cells usually do not grow well in media containing fructose but not glucose due to low or possibly no expression of GLUT5 transporter protein. Cells express GLUT5 show a good lactate metabolism profile when fructose is present, indicating an increase in lactate consumption efficiency (Wlaschin and Hu, 2007; Le et al., 2013; Wilkens and Gerdtzen, 2015). In 2013, Le et al. used the ability of the promoter of the thioredoxin-interacting protein (TXNIP) gene to drive GLUT5 expression in late cell culture by driving the expression of the GOI as the cells grow, allowing glucose to stimulate cell growth in early culture and later shifting the cells to metabolize fructose, which facilitates the overall cellular metabolic balance (Le et al., 2013).

By stably expressing both GLUT5 and pyruvate carboxylase (PYC), energy metabolism was improved, and lactate production was reduced, resulting in increased cell density and prolonged cell life span. Cellular metabolic flux analysis showed that CHO cells subjected to double gene editing had higher metabolic fluxes in glycolysis and TCA cycles, were able to consume more fructose, and maintained higher cell density (Wilkens and Gerdtzen, 2015).

## Pyruvate Production, Transport, and Consumption

During the growth and protein expression of CHO cells, the culture medium can provide various nutrients to the cells. Among them, some carbohydrates, lipids, and amino acids can be metabolized by the cells to produce energy. These substances can be converted to each other to maintain the metabolic balance of the cells, and pyruvate plays a very important role in this conversion process. For example, the metabolism of amino acids such as alanine and glycine, lipids, such as glycerol and fatty acids, and various hexoses are all related to the metabolism of pyruvate. Especially for the TCA cycle, pyruvate is one of the most important metabolic substrates. In addition, during the production, transport, and consumption of pyruvate, the generated substances, such as nicotinamide adenine dinucleotide (NADH), are essential for the regulation of cellular metabolism and influence the efficiency of metabolism of various substances. Notably, pyruvate is also the only source of lactate, a waste product of cellular metabolism, and therefore, the study of pyruvate metabolism becomes an important part that cannot be bypassed when solving problems such as lactate production and accumulation. Since the processes of pyruvate production, transport and consumption involve multiple metabolites and key enzymes, various GOIs are available to optimize the metabolism of pyruvate.

### Production

Pyruvate is the main product of the glycolytic process in CHO cells, and the concentration of intracellular pyruvate is mainly influenced by its production rate. If production rate exceeds consumption rate, a higher glycolytic flux leads to the accumulation of pyruvate. The rapid production and accumulation of pyruvate lead to the accumulation of lactate (Wilkens et al., 2011) and alanine (Ma et al., 2009) early in the course of fed-batch cultures. This was verified in the metabolic profiling data (Zhang et al., 2004; Sheikholeslami et al., 2014). One idea is to slow down glycolysis to avoid pyruvate accumulation, which will inevitably affect the normal metabolism, growth, and protein expression of CHO cells. For example, inhibition of GLUT1 (Paredes et al., 1999) expression will lead to a decrease in both cell growth rate and maximum cell density (Altamirano et al., 2013). Another idea is to increase the rate of glycolysis and simultaneously promote pyruvate depletion using gene editing techniques. However, feedback inhibition exists for key enzymes in glycolysis, such as hexokinase, phosphofructokinase (PFK), and pyruvate kinase; for example, PFK can be inhibited by ATP, low pH, and lactate, whereas hexokinase is inhibited by glucose 6-phosphate (Halestrap and Price, 1999; Costa Leite et al., 2007), suggesting that it is difficult to genetically edit the glycolytic pathway.

The site of pyruvate production is primarily the cytoplasm, but its metabolism is in the mitochondrial matrix. Although pyruvate can freely cross the outer mitochondrial membrane (through pores or non-selective channels), it needs to cross the inner membrane and enter the mitochondrial matrix with the help of the mitochondrial pyruvate carrier (MPC), which participates in the TCA cycle, gluconeogenesis, and the metabolism of lipids and amino acids to provide energy to the organism (Vanderperre et al., 2015). Therefore, MPC can regulate the energy metabolism of the organism by regulating the flux of pyruvate into the mitochondrial matrix.

### Transport

MPC are pyruvate transport proteins located on the inner mitochondrial membrane, which were identified by Papa and Halestrap (Papa et al., 1971; Halestrap, 1975) in the 1970s and further characterized in mammals in 2012 (Brivet, 2003; Bricker et al., 2012; Herzig et al., 2012). In mammalian cells, MPC is a dimeric complex consisting of two subunits, MPC1 and MPC2, and loss of activity of either subunit results in loss of activity of the MPC complex. It has been demonstrated that deletion or transcriptional repression of MPC1 results in defective mitochondrial pyruvate uptake and accumulation of glycolytic intermediates (Bricker et al., 2012; Herzig et al., 2012). Therefore, it can be assumed that the presence of MPC is a key factor in determining the occurrence of pyruvate transport or accumulation. Researchers (Bulte et al., 2020) constructed CHO cell lines stably overexpressing two subunits of the MPC complex to facilitate pyruvate entry into mitochondria and participate in the aerobic oxidation. Compared to controls, CHO cells overexpressing MPC produced up to 50% less lactate, had increased specific cell growth rates and maximum live cell densities, and transiently expressed a 40% higher maximum concentration of two recombinant model proteins, alkaline phosphatase, and monoclonal antibody. The results of the cell metabolism model showed that overexpression of MPC increased the metabolic flux of pyruvate across the mitochondrial membrane and promoted cell growth. However, the accelerated cell growth resulted in faster nutrient consumption in the medium, making it difficult to maintain a high-density culture of cells. Therefore, fed-batch culture is an optional method to develop the potential of this cell line. Regarding lactate production, the MPC overexpressing cell line showed faster lactate production during the first 12 h of culture, which was associated with higher growth and glycolysis rates. However, the rate of lactate production in this cell line rapidly decreased and was significantly lower than that of the control group, and the point in time at which lactate consumption began was earlier than the control group, a metabolic feature largely attributable to the translocation of pyruvate from the cytoplasm to the mitochondria.

### Consumption

Pyruvate consumption is inseparable from the metabolism of lactate, nearly two-thirds of pyruvate generated by glycolysis is converted to lactate (Jimenez et al., 2019). Lactate dehydrogenase (LDH) catalyzed lactate production and consumption is a reversible reaction; therefore, the LDH-catalyzed reaction in CHO cells is often in equilibrium, and it is the relative concentration of the substrate for this reversible reaction that is the key factor in determining the direction of the reaction (Quistorff and Grunnet, 2011). For example, elevated levels of pyruvate or NADH favor the production of lactate for the forward reaction, whereas elevated levels of lactate or NAD^+^ favor the consumption of lactate for the reverse reaction. Typically, the ratio of NAD^+^/NADH in the cytoplasm is ∼700:1, whereas the ratio of lactate/pyruvate is ∼20:1 (Wilkens and Gerdtzen, 2015). Changing the product/substrate ratio can shift the equilibrium position, which can drive the reversal of the LDH reaction, leading to lactate depletion. The production of lactate is limited to LDH catalysis, and the extent of its accumulation depends almost entirely on this reversible reaction, but pyruvate can participate in multiple metabolic pathways, suggesting that various factors can influence its concentration. When pyruvate is consumed by enzymes other than LDH, there is a large reduction in intracellular pyruvate, which also upsets the balance of the LDH reversible reaction, making the reaction favorable to lactate consumption. Therefore, editing key genes as GOIs for CHO metabolic engineering in other depletion pathways of pyruvate could provide a new idea to solve problems, such as lactate accumulation.

In CHO cells, the main metabolic route for pyruvate consumption other than lactate production is through MPC into the mitochondria, and acetyl coenzyme A is produced by the action of the dehydrogenase complex. Therefore, to promote pyruvate consumption, GOIs for editing in this metabolic route is warranted. For example, in the early stages of CHO cell culture, the pyruvate dehydrogenase (PDH) complex may be inactivated by pyruvate dehydrogenase kinase (PDK) phosphorylation, resulting in the inability to metabolize pyruvate (Jeong et al., 2006). Therefore, knockdown of PDK can increase the activity of PDH (Zhou et al., 2011), which is beneficial for directing pyruvate to acetyl coenzyme A, promoting the oxidation of pyruvate, and solving the problem of lactate production due to the massive accumulation of pyruvate. Reducing the expression of LDH-A alone can also reduce lactate production. However, despite a 45–79% decrease in lactate levels, specific productivity (Qp) and protein production did not increase significantly, suggesting that the knockdown of LDH-A alone in CHO cells is insufficient to effectively increase Qp and protein production (Kim and Lee, 2007). In contrast, simultaneous inhibition of LDH-A expression with PDK can successfully reduce lactate formation and simultaneously increase volumetric productivity (Zhou et al., 2011). In addition, pyruvate can be directly carboxylated to oxaloacetate catalyzed by PYC, which reduces pyruvate content while directly replenishing the carbon source for the TCA cycle and accelerating the cycle rate. It has been demonstrated that CHO cells overexpressing yeast pyruvate carboxylase (PYC2) showed an approximately fourfold reduction in lactate accumulation and a 70% increase in monoclonal antibody production compared to controls (Gupta et al., 2017).

## Aerobic Oxidation

In a typical oxygen-depleted environment, the energy production rate of mammalian cells is approximately 2.5–4.5 pmol ATP/cell/h. Cells in the growth phase require approximately 30% more energy than in the resting phase (Mulukutla et al., 2010). Aside from the energy required to maintain cell growth and proliferation, cells require more energy to synthesize and transport recombinant proteins. According to researchers’ calculations (Akashi and Gojobori, 2002; Seth et al., 2006), cells need at least approximately 17,000 ATP to synthesize a typical IgG. When the IgG specific production of CHO cells is 20 pg/cell/d, cells require 0.1 pmol ATP/cell/h (Dhami et al., 2018). TCA cycle is the main pathway of ATP production (Vander Heiden et al., 2009; Altamirano et al., 2013; Young, 2013). Therefore, TCA cycle-related gene editing is essential to improve recombinant protein expression in CHO cells.

### TCA Cycle

The TCA cycle is the metabolic center of mammalian cells. To determine the key metabolic responses of the TCA cycle in CHO cells, researchers (Dhami et al., 2018) transiently downregulated the expression of each TCA cycle gene in CHO cells using siRNA and examined its effects on cell growth and energy production. The results showed that the silencing of at least four TCA cycle genes was detrimental to the growth of CHO cells. Notably, the reaction catalyzed by mitochondrial aconitase (ACO2) is one of the key points of the TCA cycle in CHO cells. Cell growth was significantly decreased (*p* ≤ 0.0001) after 48–72 h of aconitase silencing using siRNA alone, demonstrating that the downregulation of aconitase genes has the most lethal effect among TCA cycle genes. Aconitase catalyzes the reversible conversion of citric acid to cis-aconitic acid and then cis-aconitic acid to isocitric acid (Krebs and Holzach, 1952). In mammalian cells, aconitase has a higher activity rate than other TCA cycle reactions, and it maintains the balance between citric acid, cis-aconitate, and isocitric acid (Costello and Franklin, 2013). Gene expression analysis and metabolic profiling of CHO cells with silenced aconitase gene showed that downregulation of aconitase gene expression caused oxidative stress and significantly reduced ATP and NAD production. High levels of oxidative stress reduce protein production, so amino acids such as pyruvate and proline can be added during fed-batch culture to reduce oxidative stress. In addition, reduced aconitase gene expression compromised the function of the entire TCA cycle and CHO cells were unable to replenish other intermediates to maintain the normal function of this cycle. This is the first demonstration of the regulation of the TCA cycle in CHO cells by the aconitase gene. Elucidation of the critical role of the aconitase gene in CHO cells allows the gene to be applied to future cell engineering strategies for efficient expression of recombinant proteins in response to oxidative stress or to regulate the rate of TCA cycle.

### Mitochondrial Metabolism

The search for corresponding target genes for editing has proven to be effective in improving cellular metabolic capacity at various points of the CHO cell gluconeogenesis pathway with the aim of promoting metabolism. However, focusing on the mitochondria themselves may allow us to regulate energy metabolism in CHO cells more accurately and efficiently. Study of mitochondrial content in CHO cells showed no significant linear correlation between mitochondrial content and cell growth and recombinant protein synthesis (O'Callaghan et al., 2015), suggesting that mitochondrial efficiency rather than abundance plays a more important role in the specific productivity of CHO cells. Therefore, for the optimization of mitochondria in CHO cells, efforts should focus on gene editing related to mitochondrial efficiency rather than just increasing the amount of mitochondria in the cell.

After depleting miR-23 using the miR-23 sponge (m23sp) in CHO cells, the researchers found that the expression of an inner mitochondrial membrane protein called Leucine Zipper and EF-Hand Containing Transmembrane Protein 1 (LETM1) improved. Therefore, LETM1 was identified as a potential target of miR-23. Meanwhile, due to the depletion of miR-23, although the growth of CHO cells was not changed, the specific productivity was increased, leading to a three-fold increase in the secreted alkaline phosphatase (SEAP) volume productivity (Kelly et al., 2015). Further studies showed that LETM1 is a Ca^2+^/H^+^ antiporter that can channel Ca^2+^ into the mitochondrial matrix, and the elevated Ca^2+^ concentration in mitochondria can activate the rate-limiting enzymes of the TCA cycle, such as PDH and ATP synthase, and subsequently increase mitochondrial activity (Osellame et al., 2012) and increase SEAP production. Mitochondrial editing using transcription activator-like effector nucleases (MitoTALENs) and zinc finger nucleases (MtZFNs) has been successfully applied in the treatment of mitochondrial diseases and other applications, while targeted editing of CHO cell mtDNA has not been reported. Although clustered regularly interspaced short palindromic repeats (CRISPR) technology has been widely used for CHO nuclear genome engineering, it is more difficult to introduce nucleic acids such as guide RNAs into the mitochondrial matrix, which becomes a bottleneck for the application of CRISPR technology on mitochondrial engineering (Jo et al., 2015; Gammage et al., 2018).

In addition, the TCA cycle is dependent on the synthesis of NADH for energy production in mitochondria; therefore, the level of NAD^+^ is important in mitochondria as a raw material for the synthesis of NADH. In mitochondrial reactions related to glucose metabolism, NAD^+^ is involved in the TCA cycle, oxidative phosphorylation, and oxidative respiratory chain (Anderson et al., 2017), and elevated NAD^+^ levels promote the overall metabolic capacity and energy production of CHO cells. Multiple NAD^+^ synthesis pathways exist in CHO cells, among which the salvage pathway is considered to be critical in controlling intracellular NAD^+^ levels (Yaku et al., 2018). Nicotinamide phosphoribosyltransferase (NAMPT), a key enzyme regulating the salvage pathway, has elevated expression in several malignant tumor cells to meet the latter’s large energy requirements, and it has been shown that inhibition of NAMPT blocks glycolysis (Tan et al., 2013). Nicotinamide (NAM) added to the cell culture medium, and the NAM generated upon NAD^+^ depletion can be catalyzed by NAMPT to synthesize nicotinamide mononucleotide (NMN) (Mori et al., 2014; Opitz and Heiland, 2015), which in turn synthesizes NAD^+^ from NMNAT. Although NAMPT is localized intracellularly in the cytosol, NAD^+^ synthesized by the salvage pathway can be imported into the mitochondria with the help of a recently discovered mitochondrial NAD^+^ transporter protein, SLC25A51 (Girardi et al., 2020; Kory et al., 2020; Luongo et al., 2020), thus suggesting that overexpression of NAMPT and SLC25A51 in CHO cells would be a worthwhile approach to try, although it has not been reported. Besides, nicotinamide mononucleotide adenyltransferase (NMNAT) is the only enzyme responsible for NAD^+^ production (Croft et al., 2020) and is a rate-limiting factor in the NAD^+^ synthesis pathway, as is NAMPT. It has been demonstrated that overexpression of nicotinamide/nicotinic acid mononucleotide adenyltransferase 1 leads to a significant increase in total cellular NAD^+^ content (Croft et al., 2018) and regulates the concentration of NAD^+^ in relation to ATP (Pinson et al., 2019). In 2009, NMNAT3 overexpressing mice were constructed by microinjection of cDNA of NMNAT3 protein into mouse oocytes (Yahata et al., 2009), and NMNAT3 is thought to be localized in the mitochondria (Berger et al., 2005). The results showed increased levels of NAD^+^ in mitochondria and enhanced mitochondrial energy metabolism in mice (Gulshan et al., 2018). This experiment has important implications for the optimization of mitochondrial metabolism in CHO cells and provides new ideas for metabolic engineering of CHO cells.

## Metabolic Models

Establishing metabolic models of mammalian cells has been hampered by the complexity of cell structure, differences in media composition and manipulation, and parameters during culture. However, with the expansion of the coverage of histological technologies and the improvement of the reliability of histological data, in 2016, researchers were able to systematically study CHO cell metabolism for the first time, they successfully established a genome-scale CHO metabolic model, iCHO1766 (Hefzi et al., 2016). The model contains 6,663 metabolic reactions, 4,456 metabolites, and 1,766 metabolic genes. Subsequently, genome-scale metabolic models for specific cell lines (e.g., CHO-K1, CHO-S, and CHO-DG44) were established based on the iCHO1766 model (Hefzi et al., 2016), we summarized and listed in Table 2 the characteristics of these models containing detailed parameters for each model, and we enumerated the organelles involved. A genome-scale metabolic network model (GEM) is a model that summarizes and connects all the data on genes, proteins, and cellular metabolism involved in the metabolism of a particular cell (O'Brien et al., 2015). Researchers can use reliable metabolic models to perform virtual experiments in a computer in a fast and inexpensive manner (Fouladiha and Marashi, 2017; Gu et al., 2019). GEMs can predict the metabolic state of a cell under specific growth conditions and is a powerful tool for cell biology and metabolic engineering (Zhang and Hua, 2015). Like other mammalian cell lines, the experimental manipulation and culture of CHO cells are both expensive and time-consuming. A reliable metabolic model of CHO cells can be used as a platform for computational analysis of cellular metabolism to predict experimental results and reduce the possibility of erroneous experimental results for the metabolic engineering of CHO cells, assisting in the selection of GOIs for metabolic engineering and prediction of the metabolic profile of cells after gene editing. For example, GEMs can be applied to study the effects of gene expression changes on metabolic pathways, cell growth, protein biosynthesis, and by-product secretion (Lewis et al., 2012). Moreover, CHO cell metabolic models can also help provide optimization strategies for culture media and experimental strategies for genetic engineering to improve recombinant protein production (Calmels et al., 2019; Traustason et al., 2019; Fouladiha et al., 2020). In addition, the ability of metabolic models to be integrated with histological data is another advantage (Hyduke et al., 2013; Kildegaard et al., 2013; Richelle et al., 2019a; Lakshmanan et al., 2019). For example, transcriptomic and proteomic data can be mapped onto models to infer the physiological properties of cells (Schaub et al., 2012; Richelle et al., 2019b).

**TABLE 2:** Cell-line-specific genome-scale metabolic models (Hefzi et al., 2016).

Name	Organelles	Metabolites	Reactions	Genes
iCHO1766	Cytosol, extracellular space, Golgi apparatus, intermembrane space of the mitochondria, lysosome, mitochondria, nucleus, endoplasmic reticulum, peroxisome	4,456	6,663	1,766
iCHO-K1		2,773	4,723	1,298
iCHO-S		2,760	4,683	1,273
iCHO-DG44		2,750	4,526	1,132

The simulation scope and capability of metabolic models can limit the application of CHO cells in the industrial production of RTPs. To obtain more reliable and accurate results, metabolic models need to be updated regularly to cover the latest research advances in molecular and biochemical fields (Schinn et al., 2020; Yeo et al., 2020). Researchers (Fouladiha et al., 2021) have made an in-depth addition to the iCHO1766 model by adding several genes, metabolites, and metabolic pathways, enabling it to analyze and predict a wider range of metabolic reactions and improving the accuracy and reliability of predictions. Metabolic gene editing for CHO cells can effectively optimize the metabolism of the cells. However, to further increase the protein production, the simultaneous pairing of expression vector, medium composition, and culture condition optimization must be considered so as to enable CHO cells to function as RTPs’ hosts and maximize protein production in a shorter time and at the lowest cost.

## Conclusion

In the past few years, we have witnessed the progress of the CHO cells in many aspects. Among them, the importance of optimizing the CHO cell line as the host cell for protein expression cannot be overstated. Since the carbohydrate metabolism of the cells directly determines whether there is enough ATP to support cell proliferation and protein expression, the engineering of carbohydrate metabolism in CHO cells requires much deeper study. Researchers identified and overexpressed or down-expressed several key enzymes in three aspects: enabling fructose and galactose to replace glucose as the start of glycolysis; solving the problem of difficult translocation of pyruvate in the cytoplasm into the mitochondria for consumption; and increasing the efficiency of mitochondrial ATP production, which enables CHO cells to maximize energy generation with minimal carbohydrate utilization and suppress the accumulation of metabolic waste. Furthermore, the optimization of cellular metabolism is also directly reflected in the increase of protein production. However, further research is still warranted. For example, the latest metabolic studies such as NAMPT, NMNAT, and SLC25A51 have not been applied to improve protein expression, and the latest CRISPR technology still needs a breakthrough, which is expected to achieve direct mitochondrial genome editing. Big data and multi-omics technologies are also beneficial to provide new research directions and research ideas, assisting in the comprehensive analysis of the metabolic condition of the edited cells for metabolic engineering, effectively using these tools will be the key to improving the research efficiency. It should be noticed that most metabolic engineering studies are still at the basic research level, which have the potential to be applied in industrial production. And many excellent cell engineering studies have been applied to improve protein yield such as the genomic deletion/inactivation of the dihydrofolate reductase gene which paved the way for an economical utilization of CHO cells for biopharmaceutical manufacturing (Wurm et al., 2011). Finally, CHO cells integrated with all the research achievements will become a better mammalian recombinant protein production platform.
